# Shared toilet users’ collective cleaning and determinant factors in Kampala slums, Uganda

**DOI:** 10.1186/1471-2458-14-1260

**Published:** 2014-12-12

**Authors:** Innocent K Tumwebaze, Hans-Joachim Mosler

**Affiliations:** Department of Psychology, University of Zurich, Zurich, Switzerland; Department of Environmental Social Sciences, Eawag, Swiss Federal Institute of Aquatic Science and Technology, Überlandstrasse 133, P.O Box 611, 8600 Dübendorf, Switzerland

**Keywords:** Collective cleaning, Shared toilets, Slums

## Abstract

**Background:**

Dirty shared toilets are a health risk to users in urban slum settlements. For health and non-health benefits among users of shared toilets to be guaranteed, their cleanliness is important. The objective of this study was to investigate the cleanliness situation of shared toilets in Kampala’s slums and the psychological and social dilemma factors influencing users’ cleaning behaviour and commitment by using the risks, attitudes, norms, ability and self-regulation (RANAS) model and factors derived from the social dilemma theory.

**Methods:**

We conducted a cross-sectional study in three slums of Kampala between December 2012 and January 2013. Data were collected from 424 household respondents that were primarily using shared toilets. Semi-structured questionnaires administered through face-to-face interviews were used in data collection. Linear regression was done for the multivariate analysis to test for the association between respondent cleaning behaviour and a combination of RANAS and social dilemma predictors.

**Results:**

Out of 424 respondents interviewed, 44.3% reported cleaning the shared toilet daily, 34.4% cleaned once or several times a week, 1.4% cleaned every second week, 5.4% cleaned once or several times a month and 14.4% did not participate in cleaning. The main RANAS factors significantly associated with respondents’ cleaning behaviour were: attitudinal affective belief associated with cleaning a shared toilet (β = −0.13, P = 0.00) and self-regulating factors, such as coping planning (β = 0.42, P = 0.00), commitment (β = 0.24, P = 0.00), and remembering (β = 0.10, P = 0.01). For social dilemma factors, only the social motive factor was statistically significant (β = 0.15, P = 0.00). The R square for the linear model on factors influencing cleaning behaviour was 0.77 and R square for factors influencing cleaning commitment was 0.70.

**Conclusion:**

The RANAS factors provide a more robust understanding of shared toilet users’ cleaning behaviour than social dilemma factors. Self-regulating factors and changing the negative affective cleaning feelings are shown to be very important for interventions to increase shared toilet users’ collective participation in their cleaning. In addition to RANAS, social dilemma factors have an important influence on slum residents’ commitment to clean their shared toilets.

**Electronic supplementary material:**

The online version of this article (doi:10.1186/1471-2458-14-1260) contains supplementary material, which is available to authorized users.

## Background

It is estimated that 2.5 or more billion people globally lack access to improved sanitation facilities [1]. This sanitation deficit continues to leave the public exposed to a wide range of faecal contaminants responsible for a multitude of diseases, especially in densely populated slums [2]. Estimates show that 4.2% or more of annual global mortality would be prevented if all people had access to safe drinking water, reliable sanitation and decent hygiene practices [3, 4]. While some people lack total access to sanitation infrastructure, for others, it is a question of access to clean sanitation facilities. Using a dirty toilet exposes a user to the risk of contracting diseases, such as diarrhoea, and other intestinal and respiratory infections. The challenge of cleanliness is most prevalent in urban slums where several families share limited toilet facilities, for example more than 10 families sharing one toilet stance (room) [5–7]. For cleanliness of the shared toilets to be guaranteed, it is imperative that user families are cooperative and collectively engage in their cleaning.

While there is substantial research around sanitation and its linkage to a wide range of preventable diseases [3, 4], evidence on the cleaning behaviour of shared toilet users is still inadequate. More researchers and practitioners need to explore this area, which is fundamental to public and environmental health, especially in low income urban areas. We argue that performance of a behaviour, such as individual cleaning of a shared toilet, can be explained largely by psychosocial determinants and understanding the influence of social dilemma factors. The psychological determinants are itemized into five factor blocks in the RANAS model of behaviour change – one of the few models applicable to a wide range of water, sanitation and hygiene practices and interventions [8]. The model synthesizes different social and health psychology theories and models and provides a structured approach for assessing, understanding, and explaining human behaviour as well as designing, implementing, and evaluating behavioural change-related interventions. The five conceptual block factors, each having a set of measurable variables include: risks, attitudes, norms, ability and self-regulating factors.

Risk factors [9] relate to a person’s perceived vulnerability of contracting a disease, severity and consequences associated with the disease if contracted, and factual knowledge on disease exposure agents and how they can be prevented [10].

Attitudinal factors indicate a person’s inclination to respond to a behaviour with some degree of liking or disliking the behaviour [11]. Attitudinal factors can be categorized into instrumental, which are outcome expectancies (e.g. beliefs on costs in terms of money, time, effort and benefits associated with a desired behaviour and [7, 12] and affective beliefs, which are feelings developed from thinking about a behaviour or its performance [13–15].

Normative factors constitute descriptive norms, which reflect perceptions on behaviours typically performed by others, and injunctive norms, which show perceptions on behaviours typically approved or disapproved by people an individual considers important in their lives [16].

Ability factors reflect a person’s confidence and belief to perform a behaviour [16, 17]. Performance of a desired behaviour also needs a person to have traits of positive self-efficacy, which means abilities to organise and execute courses of action required to manage potential conditions, such as dealing with barriers that arise during the performance of the behaviour and recovery from setbacks [18]. One major precondition of ability factors is action knowledge an assumption that one knows how to perform the desired behaviour [19].

Self-regulating factors take precedence after the behaviour is in place and being performed but needs sustainability over time [18, 20]. To consistently perform a desired behaviour, an individual should have the ability to manage conflicting goals and distracting situations [21]. Self-regulating factors involve action control (strategy for a continuous standard evaluation of on-going desired behaviour) [18], action planning (perceived thoughts on how to set up the behaviour and remembering and commitment to perform the desired behaviour [22].

Each of the above RANAS model factors can be assessed using a structured questionnaire and may involve a set of variables for each factor [8].

In contrast to the RANAS factors, social dilemmas are conflict situations characterised by decision-making processes, with most individuals making decisions that foster self-interests rather than those of groups to which they belong [23, 24]. Yet, individuals would be better off making decisions that benefit the whole group [25]. For instance, in the case of cleaning shared toilets, if all users of the shared toilet decided not to clean it, they would all receive lower payoffs, such as being exposed to the risk of diseases from the dirty toilet. Thus, the interest of integration of social dilemmas in this paper is on users of shared toilets’ cooperation, collective action, and commitment in their cleaning [26]. As reported in some studies, proper hygiene practice is important to avert the risks of contracting diseases associated with unhygienic situations, such as using dirty toilets [4, 27]. Sanitation research from the social dilemma perspective is still limited. Only a few studies were found that indirectly looked at the influence of some social dilemma factors, such as social norms on adoption of health behaviours [28, 29].

In this study, we investigate the influence of social dilemma factors, such as group size, social identity, social motives, social norms, behaviour of others and communication on collective cleaning behaviour of shared toilet users.

First, we considered the size of the groups since this has been reported to have an influence on individuals’ cooperation in social dilemma situations. Studies have shown that the degree of cooperation declines with an increase in the size of the groups [30, 31]. This argument is also evidenced in different sanitation studies that have shown the linkage between dirty shared toilets and the high number of user families [6, 32].

Second, social identity is reported to positively influence cooperation among individuals. For example, in groups or in this case among users of a shared toilet to participate in cleaning, a sense of belonging or oneness as users of the toilet has a positive effect [25, 33].

Third, social motive factors involve individual consideration of other people’s benefits while making individual decisions [31]. Social motives among users of shared toilets could be manifested in their selfless cooperation in maintaining the cleanliness of shared toilets [34, 35].

Fourth, social norms (shared beliefs and values that guide the way people behave or relate with each other) are reported in a number of studies as key in promotion of cooperation in resolving social dilemmas [24, 36]. For example, social norms are important in the promotion of health behaviours, especially in the field of sanitation and hygiene [28].

Furthermore, the behaviour of individuals as manifested in their decisions on whether to cooperate or not in social dilemma situations is influenced by their interpretations and observations of the behaviour of other persons in the same setting [37]. Individuals are more likely to develop a cooperative behaviour if most of the others are cooperative [38].

Lastly, communication has a cardinal influence in promoting cooperation and resolution of conflict situations, especially through face-to-face communication [39, 40]. The importance of communication and using appropriate communication channels has also been of interest in sanitation and hygiene studies [27, 41].

The objective of this study was to investigate the cleanliness situation of shared toilets in Kampala’s slums and the psychological and social dilemma factors influencing users’ collective cleaning behaviour and commitment. In regard to the operationalization of the RANAS model in understanding water and sanitation related-behaviours, studies have shown its effectiveness, such as in uptake of solar water disinfection (SODIS) [15] and consumption of fluoride-free water [42]. However, this is the first study applying RANAS and social dilemma factors to understand the cleaning behaviour of shared toilet users. While shared sanitation facilities take a broad spectrum of communal, public, and specific household shared facilities [43], our study concentrates on the latter.

## Methods

### Study origin and design

This cross-sectional study, conducted between December 2012 and January 2013, focused on users of shared toilets in three slums in Kampala, Uganda. It builds on the user-driven sanitation survey conducted in 2010 that assessed the sanitation situation in 50 slums of Kampala. Kampala is the capital city of Uganda and is divided into five municipal councils (Central, Makindye, Kawempe, Nakawa and Rubaga). The political administration and delivery of services in the city are the responsibilities of the Kampala Capital City Authority (KCCA). Slums dominate most of the city’s suburbs which support over 60% of the population in Kampala [7, 44]. The findings from the 2010 survey showed that more than half of the 1500 interviewed respondents were using dirty toilets [44]. Most of the dirty toilets were those used by more than one family [7]. Thus, this study provides further assessment on the cleanliness of shared toilets and factors influencing users’ collective cleaning behaviour.

### Target respondents and sampling procedure

We interviewed only users of shared toilets in three slums that were part of the 50 slums of Kampala surveyed in 2010 that had most dirty toilets. These slums were located in the three municipal councils of Makindye (Lufula), Kawempe (Mulago III), and Rubaga (Kironde). Lufula and Kironde slums are located in low-lying areas with a high water table and are often prone to floods. Economically, Lufula slum is known for metal fabrication while Kironde has mainly small glossary shops selling retail items, as such as food stuffs. In contrast, Mulago III is located on a high ground and is close to Mulago hospital, the main referral hospital in the country. The main activities in this slum are also small glossary shops with a market within the proximity due to the presence of the hospital. However, while most of the sanitation facilities in Mulago are simple pit latrines, Lufula and Kironde have improved ventilated pit latrines because of the high water table. All households that were using a shared toilet in the study areas were included in the sample. Because of the variation in the size of the zones and number of households sharing toilets, 200 household respondents were interviewed in Kironde, 127 in Lufula and 97 in Mulago III. We defined shared toilets as facilities used by more than one family and by users mostly geographically defined or known to each other [43]. Users of private toilets (only one family using a toilet room) or public toilets (toilets open to all, with a caretaker or often users having to pay per visit) were excluded from this survey. Our target respondents were household individuals that shared a toilet room. In each household, only one person was interviewed, mainly the household head or spouse. An eligible participant was only interviewed upon giving consent. However, exceptions occurred during data collection where respondents other than household heads or spouses were interviewed because it was not possible to have appointments with the target respondents during the study period. In this case, other household respondents aged 18 years and above were interviewed if found at home. All in all, a total of 424 respondents using 41 toilet facilities were interviewed.

### Data collection and analysis

Semi-structured questionnaires were used to collect data on socio-demographic factors and collective cleaning behaviour of the shared toilet users. The questionnaires were administered through face-to-face interviews. The interviews were conducted in the local native language (Luganda), which is the most spoken language in the study areas. Questionnaire translation and back translation was done by a professional translator, and translations were verified with other local language experts and re-verified during interviewer training and pretesting of the questionnaire. Six research assistants were recruited and taken through a series of training prior to actual field work to provide support in data collection. The questionnaire was pretested and revised before actual data collection to ensure quality. The questionnaire items included respondents’ socio-demographic factors, type of shared sanitation facility, behavioural psychological factors and social dilemma factors (see Additional file 1).

Collected data were regularly checked by the field supervisor and the principal investigator to ensure quality and completeness of the questionnaires. The Software Package for Social Sciences (SPSS) was used in the analysis of collected data. Frequencies, percentages, means and associations were generated through the various univariate, bivariate, and linear regression analyses. All RANAS and social dilemma predictors that significantly related to the cleaning behaviour of users of shared toilets at bivariate analysis were included in the linear regression model at multivariate analysis (an alpha level < 0.05 was used to determine statistical significance). The main dependent variable was cleaning behaviour, measured by shared toilet users’ self-reported cleaning frequency on a five-point scale (1 = never, 2 = once/several times a month, 3 = every second week, 4 = once/several times a week, 5 = everyday/more often). To get these responses, a respondent was asked how often he/she cleans the shared toilet.

### Ethical approval and participant informed consent

This research was conducted in strict compliance with the ethical principles of the American Psychological Association and the Declaration of Helsinki. Ethical research approval for this part of the research was obtained from Ethical Review Boards of the University of Zurich, and the Uganda National Council of Science and Technology. This research is part of the overall investigations on household demand and behaviour for improved sanitation in Kampala urban slum settlements.

In addition, only adults (mainly household heads or spouses) were interviewed in this study. Written informed consent for participation in this study was obtained from the participants and only those who consented were interviewed.

## Results

The socio-demographic characteristics of the respondents are shown in Additional file 1. The majority of the respondents were female (75%), the mean age of the respondents was 31 years (range 18–75 years), and the majority interviewed were tenants (91.5%).

The mean number of people living in respondents’ households was about 4-persons (3.55) per household (range 1–30).

### Cleanliness of shared toilets

Overall, over half of the shared toilets were reported clean (Table 1); however, interviewer observations showed that more shared toilets were very dirty than what was reported by the interviewees. There was a statistically significant Pearson correlation coefficient (P = 0.01) between interviewee perceived cleanliness and observed cleanliness by the interviewers.

**Table 1:** **Perceived and observed cleanliness**

Variables	Frequency		Percentage	
	Perceived	Observed	Perceived	Observed
Not dirty at all	271	225	63.9	53.8
A little bit dirty	44	41	10.4	9.8
Quite dirty	13	22	3.1	5.3
Dirty	65	59	15.3	14.1
Very dirty	31	71	7.3	17.0
Total	424	418	100.0	100.0

The reasons mentioned by respondents (n = 271) whose shared toilets were clean mainly related to the issue of cleaning them daily (62%) and cooperation (34.3%); other reasons (accounting for 3.7%) included every user household having a cleaning day, easy to clean toilet, few users, good toilet floor, and lockable toilet.

On the other hand, respondents (n = 153) whose toilets were dirty mainly attributed it to a large number of user families (40.9%) and lack of cooperation (30.2%); other reasons included bad use by some tenants (9.4%), misuse by children (5.4%), toilet almost full (3.4%), toilet full (2.7%), toilet having maggots (2%), not yet cleaned (2%), and misuse by outsiders (2%). Excreta on the walls and floor of the toilet room accounted for 2.1% of the respondents.

Cleaning of the shared toilets was largely attributable to gender. More than a third of the respondents (73.1%) reported that females were mainly responsible for the cleaning of shared toilets. About 15% of the respondents mentioned that males were mainly responsible for cleaning, and 9.9% of the respondents reported that both males and females were responsible for cleaning. Only 2.1% of the respondents mentioned that nobody was responsible for cleaning in their households.

The four main features reported by respondents for a clean toilet room were absence of excreta on the toilet floor (71.2%), no smell (64.2%), no flies (46%), and a dry toilet floor – not soaked with urine (41.3%). More information is shown in Table 2.

**Table 2:** **Respondents’ understanding of a clean toilet and what is used in cleaning**

Variables	Frequency (N = 424, multiple responses)	Percentages
**Perceived understanding of a clean toilet**		
No faeces	302	71.2
Toilet does not smell	272	64.2
Toilet room has no flies	195	46.0
Floor soaked with urine	175	41.3
Faeces on toilet walls	30	7.1
Toilet room has no maggots	27	6.4
Toilet hole cover lid available	20	4.7
Toilet ventilated	5	1.2
**Cleaning items**		
Water mixed with soap detergent	313	73.8
Broom	305	71.9
Plain water	65	15.3
Cleaning brush	46	10.8
Use a cleaning rag	5	1.2
Smoking it using papers	4	.9

For cleaning frequency, 44.3% of the 424 respondents reported cleaning the shared toilet daily, 34.4% cleaned once or several times a week, 1.4% cleaned every second week, 5.4% cleaned once or several times a month, and 14.4% were not involved in cleaning at all. The respondents were using mostly brooms (71.9%) and a mixture of water with detergent (73.8%) to clean (Table 2).

The improved ventilated pit latrines were the most dominant type of toilet (74.8%), followed by simple pit-latrines (14.1%) and pour flush toilets (11.1%).

A number of diseases were reportedly associated with a dirty shared toilet. Out of 424 respondents, the diseases most frequently (multiple responses) mentioned were diarrhoea (70%), cholera (58.7%), candida (41%) and dysentery (17.2%).

### Factors influencing shared toilet users cleaning behaviour

To determine the factors influencing collective cleaning of shared toilets by users, we assessed respondents’ self-reported cleaning frequency on the psychological (RANAS) and social dilemma factors using regression analysis.

### RANAS and social dilemma factors

In the first step of the linear regression, RANAS variables accounted for 75.4% of the variation in respondents’ cleaning behaviour (Table 3). The introduction of social dilemma factors in the regression model increased the variance explained by the model to about 77%, as indicated by the R square = 0.77. There was no collinearity in the regressed variables (VIF below 6). The factors that were not statistically significant to respondents’ cleaning behaviour were excluded from the hierarchical linear regression. These included the affective factor to use a dirty toilet (RANAS), social identity factors of households relationships, behaviour of others, individuals’ cleaning cooperation and individuals participating less in cleaning, and unintended non-cleaning cooperation factor of individuals who were not held responsible for toilet dirt due to their inabilities (social dilemma).

**Table 3:** **Linear hierarchical regression of respondent’s cleaning on RANAS and social dilemma variables**

Factor blocks	Variables	Unstandardized coefficients		Standardized coefficients	t	Sig.
		B	Std. error	Beta		
	Step 1					
	(Constant)	.390	.440		.886	.38
Risk factors	Vulnerability to get disease	.052	.080	.017	.656	.51
	Severity of disease	-.060	.084	-.019	-.710	.48
Attitude factors	Affective feeling - cleaning shared toilet	-.059	.015	-.126	−3.998	.00
	Instrumental - cleaning time consuming	.071	.047	.055	1.511	.13
	Instrumental - cleaning effort	.039	.040	.035	.976	.33
Norm factors	Injunctive - approval to clean	.015	.020	.023	.740	.46
	Injunctive - social pressure to clean	.017	.026	.018	.657	.51
Ability factors	Self-efficacy - cleaning difficulty	-.006	.034	-.007	-.178	.86
	Self-efficacy - cleaning schedule	-.064	.029	-.063	−2.239	.03
Self-regulation factors	Action planning - cleaning daily routine	.505	.048	.521	10.538	.00
	Remembering to clean	.139	.049	.115	2.825	.01
	Cleaning commitment	.287	.052	.287	5.505	.00
	Step 2					
	(Constant)	.331	.451		.735	.46
Risk factors	Vulnerability to get disease	.031	.079	.010	.398	.69
	Severity of disease	.023	.084	.007	.269	.79
Attitude factors	Affective feeling - cleaning shared toilet	-.060	.015	-.129	−4.055	.00
	Instrumental - cleaning time consuming	.076	.047	.058	1.610	.11
	Instrumental - cleaning effortful	.048	.040	.043	1.205	.23
Norm factors	Injunctive - approval to clean	.012	.020	.017	.576	.57
	Injunctive - social pressure to clean	-.003	.026	-.004	-.133	.89
Ability factors	Self-efficacy - cleaning difficulty	-.026	.036	-.028	-.713	.48
	Self-efficacy - cleaning schedule	-.069	.029	-.068	−2.346	.02
Self-regulation factors	Action planning - cleaning daily routine	.405	.051	.419	7.937	.00
	Remembering to clean	.118	.049	.097	2.410	.02
	Cleaning commitment	.237	.053	.237	4.462	.00
Social motive factor	Respondents cleaning more than other users	.091	.021	.146	4.247	.00
Communication factors	Talking frequency	.007	.035	.005	.191	.85
	Talking ease	.030	.033	.032	.903	.37
Perceived efficacy factors	Shared toilet users’ cleaning cooperation	.042	.036	.043	1.169	.24
	Cleanliness confidence if other users are cooperative in cleaning	-.085	.053	-.045	−1.601	.11
Group dynamics factor	Cleaning team	.057	.038	.063	1.508	.13

Step 1: Regression of cleaning behaviour on RANAS variables, N = 417, R Square = .75. Step 2: Regression of cleaning behaviour on RANAS and Social dilemma variables, N = 415, R Square = .77.

The negative, statistically significant, attitudinal affective factor associated with respondents’ cleaning of the shared toilet indicated that the more respondents dislike cleaning a shared toilet, the less they participated in cleaning. The negative, statistically significant, ability factor of a cleaning schedule indicated that respondents’ cleaning behaviour is less if their households have no cleaning roster regarding when to clean the shared toilet. On the other hand, the statistically significant self-regulating factors showed that respondents are more likely to frequently clean shared toilets if cleaning is part of their daily routine activities, it is easier to remember when to clean, and there is a cleaning commitment. Only one of the social dilemma variables was statistically significant. Respondents who believed they were cleaning more than the other shared toilet users participated more in collective cleaning, as shown by the social motive factor.

### Respondents’ cleaning commitment

As shown in Table 4, social dilemma factors accounted for 67% (R Square = .67) of the variation in respondents’ collective cleaning commitment of the shared toilets.

**Table 4:** **Linear regression of respondents cleaning commitment on social dilemma factors**

Variables		Unstandardized coefficients		Standardized coefficients	Sig.
		B	Std. error	Beta	
	(Constant)	.726	.259		.01
Social motives	Cleaning toilet more than other users	.166	.016	.338	.00
Social identity	Shared toilet users’ relations	.086	.020	.219	.00
Behaviour of others	Cleaning households	.003	.001	.072	.02
	Individual’s cooperation in cleaning	.018	.039	.013	.65
	Respondents cleaning less than other users	-.083	.018	-.162	.00
Communication	Talking frequency with other users	.081	.031	.080	.01
	Easy to talk to other users	.154	.028	.212	.00
Unintended non-cooperation	Individuals not held responsible	.049	.043	.032	.26
Perceived efficacy	Shared toilet users’ cleaning cooperation	.036	.033	.048	.28
	Cleanliness confidence if other users are cooperative in cleaning	.136	.046	.092	.01
Group dynamics	Cleaning team	.084	.033	.118	.01

N = 422, R Square = .70.

Social dilemma factors such as social motives, social identity, communication, and group dynamics, were positively related to respondents’ commitment to clean their shared toilets. Commitment was greater among respondents who believed they cleaned more than other users of the shared toilets, who positively related with other users, who easily talked with other users, and who felt they were part of a team with other users. However, while the perceived efficacy factor of household cooperation to clean shared toilets was not statistically significant, commitment was likely among respondents who had confidence that cleanliness of the shared toilets depended on the cooperation of all user households. Lastly, the behaviour of other households’ cooperation in cleaning of the shared toilets was not statistically significant. However, cleaning commitment by shared toilet users was less among respondents who reported cleaning less than the other toilet users.

## Discussion

The aim of this study was to investigate the cleanliness of household shared toilets in three urban slums in Kampala and assess the factors influencing users’ cleaning behaviour.

### Cleanliness of shared toilets

Overall, the level of cleanliness of household shared toilets in the three studied slums of Kampala was above 50%. Six of every 10 household respondents reported that their shared toilets were clean. This is moderately consistent with interviewer observations that showed five of every 10 respondents having clean shared toilets. The respondents mainly mentioned a shared toilet as clean if the toilet room had no excreta on the floor, did not smell, had no flies, and had a dry floor – that was not flooded with urine. As reflected previously on determinants of households’ cleaning intention for shared toilets [7], respondents’ perceived toilet cleanliness is reported more than is observed by interviewers. The lack of cleanliness of shared toilets is one of the key reasons why shared toilets are considered as unimproved by the United Nations Joint Monitoring Program for Water and Sanitation [1]. Indeed, a number of studies have documented the unclean situation of shared sanitation facilities in most urban informal settlements [6, 7].

Cleanliness of the shared toilets was largely dependent on users’ cleaning frequency and cooperation. Six out of every 10 household respondents reported cleaning their shared toilets on a daily basis, and cleaning cooperation among user households was reported in three of every 10 household respondents. On the other hand, dirty toilets were mainly attributed to the large number of users (four of every 10 household respondents) and lack of cleaning cooperation (three of every 10 household respondents). These findings suggest that regular cleaning and cooperation among user households is important to maintain hygienic conditions of shared toilets. In line with other studies, this study found that shared toilets were more likely to be dirty if they were being used by a big number of households [5, 32]. One of the reasons why many users of a shared toilet could lead to deterioration in its cleanliness is the diffusion of cleaning responsibilities and lack of cooperation [45].

Furthermore, we found that the cleaning of shared toilets was related to gender, with females being six times more involved in cleaning than males. This is not surprising since women are more involved in preventive health undertakings with regard to domestic hygiene [46]. The main materials reportedly used in cleaning toilets in this study were brooms and water mixed with detergent. Most of the shared toilets were ventilated.

### Factors influencing respondents’ cleaning of shared toilets

The determinants that significantly relate to the cleaning behaviour of shared toilet users are explained by RANAS and social dilemma factors. The RANAS model of behavioural change is key to understanding the cleaning behaviour of shared toilet users. This study shows that most variations in respondents’ cleaning behaviour for shared toilets can be explained by the RANAS model rather than the social dilemma. The most important of the RANAS and social dilemma factors, with high beta values, are the self-regulating factors and the social motive factor, respectively (Table 3).

Self-regulating factors, such as action planning, remembering, and commitment, significantly relate to respondents’ cleaning behaviour for their shared toilets. First, action planning is a key factor in cleaning of shared toilets by users. The respondents were more likely to report frequent participation in cleaning their shared toilet if ensuring cleanliness of the shared toilet was one of their routine activities. This finding is in agreement with that of other studies on the importance of action planning in sustained behavioural performance [18, 21]. For example, a physical activity study on whether action planning was beneficial to patients who had the intention to exercise but did not showed that patients who had been inactive but intended to exercise benefited more from planning intervention than patients without the intention or those who were already active [18]. The implication of this study finding is that action planning, as reflected in shared toilet users’ integration of cleaning as part of their routine activities, fosters control and continued performance of the cleaning behaviour.

The second self-regulating factor influencing cleaning behaviour of shared toilet users is commitment. The more that respondents were committed to cleaning their shared toilet, the more they participated in toilet cleaning. Our finding implies that people are more likely to perform a behaviour if they are committed to its performance. A study by Bandura contends that the higher the goals are that people set for themselves and their perceived efficacy, the more likely is their commitment to achieve the desired behaviour [47].

The third self-regulating factor influencing cleaning behaviour related to remembering when to perform the cleaning. The respondents who found it easy to remember when to clean were more likely to participate in cleaning than those who found it almost impossible to remember. The performance of a desired behaviour needs to be supported with prompts set by an individual to act as triggers or reminders to help remember the behaviour [8]. The implication of the study finding is that behaviour is performed more if it is easy to remember when it needs to be performed [22].

The other RANAS factor significantly associated with respondents’ cleaning behaviour is the affective factor. Respondents were less likely to clean a shared toilet if they disliked cleaning. If a behaviour is associated with emotional displeasure, the chances are low that it will be performed [13, 14]. On the other hand, positive affect is reported to have a high likelihood for one to perform or adopt a health behaviour [15]. A study on persuasion factors influencing the decision to use sustainable household treatment showed that these factors had a positive affect towards solar water disinfection (SODIS). In that study, respondents were asked how they felt about SODIS; responses ranged from very positive to very negative [15]. Thus, in settings such as slums where facilities are shared and users are responsible for their maintenance, it is important that persuasive approaches that encourage cleaning behaviour performance are promoted, such as stressing health attributes from using a clean toilet.

Last, as shown in Table 3, respondents’ social motives had a significant influence on their cleaning behaviour. We found that respondents’ cleaning was common among those who believed they were cleaning the shared toilet more than the other user households. As indicated in some social dilemma research, social motive factors are manifested when one takes the outcomes of others into account when making choices [48]. Cleaning of shared toilets in this study was mainly reported among respondents who perceived their amount of cleaning to be more or the same as others who were participating in cleaning. The implication from this finding is that promotion of cleaning as a social motive factor is important among users of shared toilets to maintain cleanliness, for example among respondents who may have toilet-going children.

### Influence of social dilemma factors on cleaning commitment

As shown in Table 4, social dilemma factors showed a great influence on respondents’ cleaning commitment for their shared toilets.

First, the social motive factor had the greatest influence on respondents’ commitment to participate in the cleaning of shared toilets. We found that respondents who believed they were cleaning the shared toilet more than the other users had more commitment than those who believed they cleaned less than the other users. A user of a shared toilet may involve more in its cleaning if he or she values using clean facilities or is aware of risks associated with having to use a dirty toilet [35]. This finding implies that promotion of values that are beneficial to all people within a given group or setting reinforces social values, which in turn may foster individual commitments in performing desired behaviours [25].

Second, respondents having a good relationship with other toilet-sharing households were more likely to commit to cleaning the shared toilet than those who viewed their relationship with other users as bad. Having a good relationship with other users promotes a feeling of togetherness and belonging which is the foundation for social identity [33]. This finding complements the study on social identity theory that states social identification leads to activities that are correspondent with the identity and support for the institutions and reinforces the antecedents for identification [33]. This is probably why group dynamics are significantly associated with respondents’ commitment to participate in cleaning shared toilets. The promotion of social identity among individuals with different ethnicities may be improved by encouraging communication among users of the shared toilets. This is further seen in our study where communication is also positively related to shared toilet users’ cleaning commitment. The more often users of shared toilets talk to each other, the more likely is their commitment to clean the shared toilets. These findings are comparable to those of other studies on the importance of communication in fostering cooperation or promotion of health behaviours [7, 40].

Finally, respondents’ commitment to clean shared toilets relate to their perceived self-efficacy. When shared toilet users are more confident in the cooperation of others in cleaning, they display a greater cleaning commitment. This finding shows that the behaviour of others can have an influence on an individual’s cleaning commitment, as seen for individuals who reported cleaning less than the other users of the shared toilet.

### Limitations and proposed future studies

This study focused on users of shared toilets in urban slums. While shared toilets vary depending on the providers, access to them, or their management, we limited our scope to only facilities where use was restricted to certain groups of people or households and who are were also responsible for their cleaning.

Interpretation and generalizability of the findings to other slum settings in Uganda as well as in other countries should be done with caution because more studies are needed to validate our findings and theories. While the RANAS model has been widely used in most water-related and hand-washing studies, none of the previous studies focused on behaviour such as the cleaning behaviour of shared toilet users. This limitation also applies to the social dilemma factors or other studies on water and sanitation. As this is a new approach for studying cleaning behaviour of shared toilet users, it would benefit from more validation studies.

Nevertheless, our findings provide a baseline which more extensive research can be conducted in the area of shared toilet users’ maintenance using the RANAS model of behavioural change techniques and items from social dilemma theory.

## Conclusions

This study has showed that RANAS and social dilemma factors are important in the assessment of health behaviours, such as cleaning behaviour among users of shared toilets in urban slums. While the RANAS factors provide a greater explanation of the factors influencing the users of shared toilets’ collective cleaning behaviour than the social dilemma factors, the social dilemma factors are equally important influencing predictors for shared toilet users’ cleaning commitment. Very important factors were self-regulating factors, affective beliefs and social motives as important predictors for cleaning behaviour, and social dilemma factors such as social motives, social identity, and communication, as important predictors for respondents’ cleaning commitment.

## Authors’ information

IKT is a PhD student of social and environmental psychology at the University of Zurich and is employed as a PhD Student at Eawag, Swiss Federal Institute of Aquatic Science and Technology. H-JM is an Associate Professor for social and environmental psychology at the University of Zurich and senior researcher and leader of the group Environmental and Health Psychology at Eawag, Swiss Federal Institute of Aquatic Science and Technology.

## Electronic supplementary material

Additional file 1: **Shared toilet user’s collective cleaning and determinant factors in Kampala slums, Uganda.** (DOCX 26 KB)
