# Supplementary material for: Shared toilet users’ collective cleaning and determinant factors in Kampala slums, Uganda
Source: BMC Public Health. 2014 Dec 12;14:1260. doi: 10.1186/1471-2458-14-1260 (PMC4295474; doi:10.1186/1471-2458-14-1260)
Supplement: Supplementary file 1 — Additional file 1: Shared toilet user’s collective cleaning and determinant factors in Kampala slums, Uganda. (DOCX 26 KB) [file 12889_2014_7372_MOESM1_ESM.docx]

**Additional Files : Shared toilet user’s collective cleaning and determinant factors in Kampala slums, Uganda**

Annex 1: Question items

1. Situational variables

| **Situational factors** | **Measurement** |
| --- | --- |
| Sex | ▪ Respondent sex [1= Male, 2= Female] |
| Age | ▪ Age in complete years [Numeric] |
| Household ownership | ▪ Do you own or rent the household you live? [1= Own, 2= Rent] |
| Years in household | ▪ How long have you been living in this house? [1= < 1 year, 2= 1 to 2 years, 3= 3 years plus] |
| Change of location plans | ▪ Do you plan to move and live in another area within a year's time from now? [1= I don't know, 2 = No, 3= Yes] |
| Religion | ▪ What is your religion [1= Catholic, 2= Protestant, 3= Muslim, 4= Others (Born again, Seventh Day Adventist)] |
| Education level | ▪ What is your highest level of formal education? [1= None, 2= Primary, 3= Secondary, 4= Tertiary] |
| Occupation | ▪ What kind of job are you employed? [1= Not employed, 2= Civil / formal employment, 3= Casual labourer, 4= Business] |
| Estimated monthly income | ▪ If employed or have some kind of work, what is your estimated monthly income? [1= < 50,000, 2= 51,000 to 100,000, 3= 101,000 to 150,000, 4= 151,000 to 200,000, 5= > 200,000, 88= do not know or no response] |
| Household population | ▪ How many people live in your household? [Numeric] |
| Children in household | ▪ How many children below 5 years are in your household? [Numeric] |
| Sanitation facility | ▪ What type of toilet does your household use? [1= Flush toilet, 2= Pour flush, 3= Ventilated Improved Pit latrine, 4= Ecosan toilet, 5= Simple Pit latrine] |
| Cleanliness satisfaction | ▪ How satisfied are you with the current cleanliness of your shared toilet? |

1. RANAS variables

| **Psychological (RANAS) factors** | |
| --- | --- |
| **Factor blocks** | **Measurement** |
| **Risk beliefs** |  |
| Vulnerability | ▪ What are the chances that you get sick if you used a dirty toilet? [1= Impossible to 5= very possible] |
| Severity | ▪ Imagine you contracted a disease like cholera, how severe would it be on your social life, household and economic situation? [1= Not severe at all to 5= very severe] |
| Factual knowledge | ▪ What diseases is one likely to contract as a result of using a dirty toilet? [Open ended] |
| **Attitudinal beliefs** |  |
| Instrumental | ▪ How time consuming is it for you to clean a shared toilet? [1= Very time-consuming to 5= not at all time-consuming ] |
|  | ▪ How effortful is it for you to clean your shared toilet? [1= Very effortful to 5= not at all effortful ] |
| Affective | ▪ How do (would) you feel to clean a toilet shared with other households? [1= I dislike it very much to 9= I like it very much] |
|  | ▪ How negative do you think it is to use a dirty toilet? [1= Not at all negative to 5= very negative] |
| **Normative beliefs** |  |
| Injunctive norms | ▪ In general, do you think most people important to you rather approve or disapprove that you clean the toilet shared with other households? [1= Very strongly disapprove to 9= very strong approval] |
|  | ▪ Do you feel a form of social pressure to clean your shared toilet? [1= Not at all to 5= very much] |
| **Ability beliefs** |  |
| Self-efficacy | ▪ How difficult is it for you to clean a toilet shared with other households? [1= Very difficult to 5= not difficult at all] |
|  | ▪ Do you or your household have any detailed schedule or roster regarding when to clean the shared toilet? [1= Not at all to 5= very much detailed schedule] |
| **Self-regulation beliefs** |  |
| Action planning | ▪ Is ensuring cleanliness of the shared toilet on the daily routine of your activities? [1= Not at all to 5= very much part of daily activities] |
| Remembering | ▪ How difficult is it to remember to clean your shared toilet? [1= Very difficult to 5= not difficult at all] |
| Commitment | ▪ Do you feel committed to cleaning your shared toilet? [1= Not at all committed to 5= very committed] |
| **Behavioural factors** |  |
| Shared toilet cleanliness | ▪ How dirty is the toilet you share with other households? [1= Very dirty to 5= not dirty at all] |
| Cleaning frequency | ▪ How often do you clean your shared toilet? [1= Never to 5= every day or more often] |

1. Social dilemma variables

| **Social dilemma factors** | |
| --- | --- |
| **Factor blocks** | **Measurement** |
| **Social motives** |  |
| Perceived cleaning frequency | ▪ Do you clean the shared toilet more or less often than the other users? [1= Much less to 9= much more] |
| **Social identity** |  |
| Households relationship | ▪ How good or bad is your relationship with the other households you share with a toilet, in terms of its cleaning? [1= Very bad to 9= very good] |
| **Behaviour of others** |  |
| Cleaning households | ▪ How many of the toilet room sharing households participate in its cleaning? [1= (Almost) nobody (0%) to 5= (Almost) all of them (100%)] |
| Individual's cooperation | ▪ How much do you think that keeping the shared toilet clean depends on your cooperation with other user households? [1= Not at all much to 5= very much] |
| Individual's cleaning | ▪ I do not clean the shared toilet more because other users do not do the same. How much do you agree with this statement? [1= I strongly agree to 9= I very strongly disagree] |
| **Communication** |  |
| Talking frequency | ▪ How often do you talk with other toilet sharing households on the way it is used or managed? [1= (Almost) never to 5= (Almost) always] |
| Talking difficult | ▪ How difficult is it to talk to other families who you share with a toilet not dirt it? [1= Very difficult to 5= not difficult at all] |
| **Noise** |  |
| Cleaning exemption | ▪ How much of the shared toilet dirt would you think is due to persons that just could not clean up or cannot be made responsible (e.g. children, elderly, sick)? [1= None (0%) to 5= (Almost) all (100%)] |
| **Perceived efficacy** |  |
| Households cooperation | ▪ How confident are you that households you share a toilet with cooperate in its cleaning? [1= Not confident to 5= very confident] |
|  | ▪ How confident are you that your shared toilet can be kept clean if all households are cooperative? [1= Not confident to 5= very confident] |
| **Group dynamics** |  |
| Cleaning team | ▪ How much do you feel as a team with other households you share a toilet in regard to its cleaning? [1= Not at all much to 5= very much] |

Annex 2: Socio-demographic characteristics

| **Variables** | **Frequency (N = 424)** | **Percentage** |
| --- | --- | --- |
| **Sex** | | |
| Male | 106 | 25.0 |
| Female | 318 | 75.0 |
| **Rental status** | | |
| Own | 36 | 8.5 |
| Rent | 388 | 91.5 |
| **Years in household** | | |
| < 1 year | 97 | 22.9 |
| 1 to 2 years | 115 | 27.1 |
| 3 years and above | 212 | 50.0 |
| **Change of location plans** | | |
| No | 302 | 71.2 |
| Yes | 53 | 12.5 |
| I don't know | 69 | 16.3 |
| **Religion** | | |
| Catholic | 137 | 32.3 |
| Protestant | 97 | 22.9 |
| Muslim | 160 | 37.7 |
| Other | 30 | 7.1 |
| **Education** | | |
| None | 32 | 7.5 |
| Primary | 162 | 38.2 |
| Secondary | 194 | 45.8 |
| Tertiary | 36 | 8.5 |
| **Employment** | | |
| None | 137 | 32.3 |
| Formal employment | 22 | 5.2 |
| Informal employment | 158 | 37.3 |
| Business | 107 | 25.2 |
| **Estimated monthly income (N = 287)** | | |
| < 50,000 | 85 | 29.6 |
| 51,000 to 100,000 | 79 | 27.5 |
| 101,000 to 150,000 | 27 | 9.4 |
| 151,000 to 200,000 | 25 | 8.7 |
| > 200,000 | 44 | 15.3 |
| Don't know / no response | 27 | 9.4 |
| **Type of household sanitation facility** | | |
| Pour flush | 47 | 11.1 |
| Ventilated Improved Pit latrine (VIP) | 317 | 74.8 |
| Simple pit latrine | 60 | 14.2 |
